# IDEDNIK syndrome: a newly recognized rare genetic disorder caused by *AP1S1* and *AP1B1* mutations

**DOI:** 10.3389/fneur.2025.1695128

**Published:** 2025-12-01

**Authors:** Rong Wu, Xingguang Luo, Xiao-Ping Wang

**Affiliations:** 1 Department of Neurology, Tongren Hospital Affiliated to Shanghai Jiao Tong University School of Medicine, Shanghai, China; 2 Department of Psychiatry, Yale University School of Medicine, New Haven, CT, United States; 3 Jiading Hospital, Shanghai First People's Hospital Affiliated to Shanghai Jiao Tong University School of Medicine, Shanghai, China

**Keywords:** IDEDNIK syndrome, MEDNIK syndrome, Wilson’s disease, *AP1S1*, *AP1B1*, copper metabolism disorder, AP-1

## Abstract

IDEDNIK syndrome (formerly MEDNIK syndrome, OMIM #609313) is a rare autosomal recessive neurocutaneous disorder characterized by dysregulated copper metabolism and multisystem involvement. The primary causative gene, *AP1S1*, encodes the σ1A subunit of the adaptor protein complex AP-1, while mutations in *AP1B1*, encoding the β1 subunit, can cause a similar phenotype. Pathogenic mutations impair intracellular vesicle trafficking, disrupting the precise sorting and transport of multiple proteins, including the copper-transporting ATPases ATP7A and ATP7B. This results in defective copper homeostasis and a clinical phenotype overlapping features of Menkes and Wilson’s diseases. Hallmark manifestations include intellectual disability, enteropathy, deafness, peripheral neuropathy, ichthyosis, and keratoderma. Laboratory findings often reveal reduced serum copper and ceruloplasmin levels, with some patients exhibiting elevated hepatic or urinary copper. Cranial MRI typically demonstrates cerebral atrophy. No curative therapy is currently available; management is multidisciplinary, focusing on symptomatic relief and complication prevention. Oral zinc acetate has been reported to improve certain clinical features and biochemical parameters. This review provides a comprehensive update on the genetics, pathogenesis, clinical spectrum, diagnosis, management, and future directions for this debilitating disease.

## 1 Introduction

IDEDNIK syndrome (formerly MEDNIK syndrome, OMIM #609313) is a severe, recently described disorder characterized by dysregulated copper metabolism and multisystem involvement. It is an extremely rare autosomal recessive genetic condition primarily caused by mutations in *AP1S1*, which encodes the σ1A subunit of the adaptor protein complex AP-1 (1). Mutations in *AP1B1*, encoding the β1 subunit of AP-1, can also cause an overlapping phenotype, sometimes referred to as a MEDNIK-like syndrome (2). The acronym “IDEDNIK” derives from its core features: Intellectual Disability, Enteropathy, Deafness, Neuropathy (peripheral), Ichthyosis, and Keratoderma.

The first case was reported by Beare JM et al. in 1972 (3), but it was not until 2005 that Saba TG et al. (4, 5) described an atypical erythrokeratodermia variant and mapped the causative locus to chromosome 7q22, paving the way for genetic characterization. In 2008, Montpetit A et al. formally identified *AP1S1* mutations as the cause and named the disorder “MEDNIK syndrome” (1). Following updates to the International Classification of Diseases (ICD), the term *mental retardation* was replaced by *intellectual disability*, prompting the updated acronym “IDEDNIK.” Reports from China remain extremely limited (6).

## 2 Etiology and pathogenesis

IDEDNIK syndrome results from biallelic mutations in *AP1S1* (7q22.1) or *AP1B1* (22q22.2). *AP1S1* encodes the σ1A subunit of adaptor protein complex 1 (AP-1) (1). AP-1 is a heterotetramer, composed of *γ*, β1, μ1, and σ1 subunits, that plays a central role in intracellular vesicle transport. It operates in the trans-Golgi network (TGN), endosomes, and lysosome-related organelles to mediate clathrin-coated vesicle formation, lysosomal protein sorting, and polarized trafficking in epithelial basolateral membranes and neuronal dendrites (5, 7). The σ1A subunit is essential for clathrin coat assembly and efficient protein cargo sorting. *AP1B1* encodes the large *β* subunit of the clathrin-associated adaptor protein 1 (AP-1) complex, a critical regulator of intracellular sorting and vesicular trafficking between the trans-Golgi network and endosomes (2). Notably, *AP1B1*-related cases can be caused by copy number variants (CNVs), which may be missed by standard next-generation sequencing and require detection via array-CGH or MLPA (2).

Mutations in *AP1S1* and *AP1B1* impair AP-1 function, disrupting trafficking of multiple proteins, including the copper-transporting ATPases ATP7A and ATP7B (8, 9). These transporters are crucial for copper homeostasis; their mislocalization mirrors pathogenic mechanisms in Menkes and Wilson’s disease. In Wilson’s disease, *ATP7B* mutations on chromosome 13q impair copper export and reduce ceruloplasmin synthesis (chromosome 3q) (8). In IDEDNIK syndrome, AP-1 dysfunction prevents copper transporters from relocating from the TGN to the plasma membrane or bile canalicular membrane--associated vesicles (10), leading to abnormal intracellular copper distribution and contributing to neurological and connective tissue manifestations.

The full pathogenic cascade remains incompletely defined, but several mechanisms have been proposed:

- Skin manifestations (ichthyosis, keratodermia): defective epithelial development due to impaired trafficking of structural and enzymatic proteins (1).
- Enteropathy: potentially from abnormal trafficking of nutrient absorption proteins, disrupted copper/zinc transport in enterocytes, and compromised barrier function (tight junction defects, loss of polarity, increased permeability) (11). Electron microscopy reveals shortened and deteriorated microvilli at the tips of intestinal epithelial cells (11).
- Peripheral neuropathy: likely from defective synaptic vesicle formation and neuronal differentiation, impairing neural network development (1).
- Hair and pigment abnormalities: copper deficiency leads to decreased tyrosinase activity, reducing melanin production, resulting in hypopigmentation of hair and skin (6, 12). Hair microscopy shows reduced pigment, pale color, and an intermittent or absent medulla, distinct from the pilli torti seen in Menkes disease (6, 12).

Together, these cellular trafficking defects explain the syndrome’s characteristic multi-system phenotype.

## 3 Clinical manifestations

IDEDNIK syndrome typically presents between birth and 12 months of age. The core features—represented by the acronym—include Intellectual Disability, Enteropathy, Deafness, Peripheral Neuropathy, Ichthyosis, and Keratoderma. Additional features such as seizures, attention deficit hyperactivity disorder (ADHD), sparse teeth, and hepatomegaly have also been reported and contribute to the phenotypic spectrum. As an autosomal recessive condition, several reported cases have been linked to a common ancestor (4, 13). The disorder exhibits significant clinical overlap with Menkes and Wilson diseases, yet is distinguished by its characteristic constellation of core features.

### 3.1 Gastrointestinal

Enteropathy is often the earliest manifestation, with congenital diarrhea being the most common presentation (11, 13). Affected patients frequently experience chronic, intractable diarrhea, vomiting, and feeding difficulties from infancy, leading to growth retardation, failure to thrive, and increased susceptibility to recurrent infections (6, 11). The enteropathy can be severe, with one reported neonate succumbing to complications at 126 days (13).

### 3.2 Dermatological

Cutaneous findings are another frequent early-onset feature. These include ichthyosis, hyperkeratosis, and dry, scaly skin (1, 4, 14). Some individuals also develop transient erythematous patches of variable size and shape (4, 14). Keratoderma can affect the palms and soles. Nail dystrophy, including partial nail loss and desquamation of the surrounding skin, has been observed (6).

### 3.3 Neurological and developmental

Neurodevelopmental involvement is a core feature. Patients exhibit moderate to severe intellectual disability (12, 14). Recent reports highlight heterogeneous neurological presentations, including epileptic seizures that may be treatment-resistant (6, 13, 15), and symptoms of attention deficit hyperactivity disorder (ADHD) in older children (6). Peripheral neuropathy, thought to emerge later in the disease course (12), manifests as hypotonia, muscle weakness, and progressive muscle atrophy (4, 14). Sensorineural hearing loss is common (12). Brain MRI typically reveals generalized cerebral atrophy (4, 12); some patients show symmetrical T2 hyperintensities in the basal ganglia, especially the caudate nucleus and putamen (12).

### 3.4 Other systemic features

Dysmorphic features reported in some patients include a high forehead, depressed nasal bridge, low-set ears, and proportionate growth retardation (4, 12). Additional manifestations may include hepatomegaly (12), sparse, brittle, and hypopigmented hair (often blonde or yellow) (6, 12), and sparse teeth, which may represent a previously under-recognized feature (6). Hepatomegaly and evidence of liver dysfunction, such as elevated aminotransferases, are also common (12).

## 4 Diagnosis and differential diagnosis

### 4.1 Clinical and biochemical diagnosis

A clinical diagnosis of IDEDNIK syndrome should be suspected in infants presenting with a combination of the core features, particularly early-onset enteropathy and ichthyosis accompanied by neurological deficits. Biochemical tests support the diagnosis:

- Copper metabolism: decreased serum copper and ceruloplasmin; in some cases, elevated hepatic and urinary copper (12–14).
- Lipid metabolism: most patients have elevated plasma very long-chain fatty acids (VLCFAs) (1, 4, 12).
- Catecholamines: some show increased plasma dopamine with reduced epinephrine and norepinephrine; urinary dopamine is also elevated (13).
- Liver function: Possible elevation of aminotransferases and bile acids, with AST/ALT ratio > 1 (12).
- Hair microscopy: can reveal trichorrhexis nodosa, irregular hair shaft diameters (12), or specific findings of reduced medulla and pigmentation, helping to differentiate from Menkes syndrome.
- Neuroimaging: brain MRI typically reveals generalized cerebral atrophy (4, 12); some patients show symmetrical T2 hyperintensities in the basal ganglia, especially the caudate nucleus and putamen (12).
- Other investigations: skeletal X-ray may detect osteoporosis (12). Abdominal ultrasound can reveal Hepatomegaly and increased hepatic echogenicity; liver biopsy may show fibrosis and/or cirrhosis (12).

### 4.2 Genetic diagnosis

Genetic testing is essential for a definitive diagnosis. Available methods include:

- Sequencing: whole-exome or whole-genome sequencing can identify sequence variants in *AP1S1* (7q22.1). Reported pathogenic variants include c. IVS2-2A > G (1), c.269 T > C (11, 13), c.186 T > G (16), c.364dupG (14), c.356_365insG (12), c.356_357insG (17), c.346G > A (11), p.Tyr62Ter (18), and the recently reported c.430-1G > A (6).
- CNV analysis: if sequencing is negative but clinical suspicion remains high, targeted copy number variant (CNV) analysis for *AP1B1* (22q22.2) using array-CGH or MLPA is recommended, as exonic deletions/duplications can cause the disease (2). A growing number of pathogenic mutations in *AP1B1* have been documented, including: c.668 T > C (19), c.667delC (20), c.2335delC (21), c.2254delC (21), chr22:29758984–29,815,476 (2), c.1852C > T (22), c.2596C > T (21, 22), c.38-1G > A (2, 23), c.430 T > C (23), c.322C > T (24), c.2374G > T (23), and c.1263C > A (21).

### 4.3 Differential diagnosis

**Figure 1:**
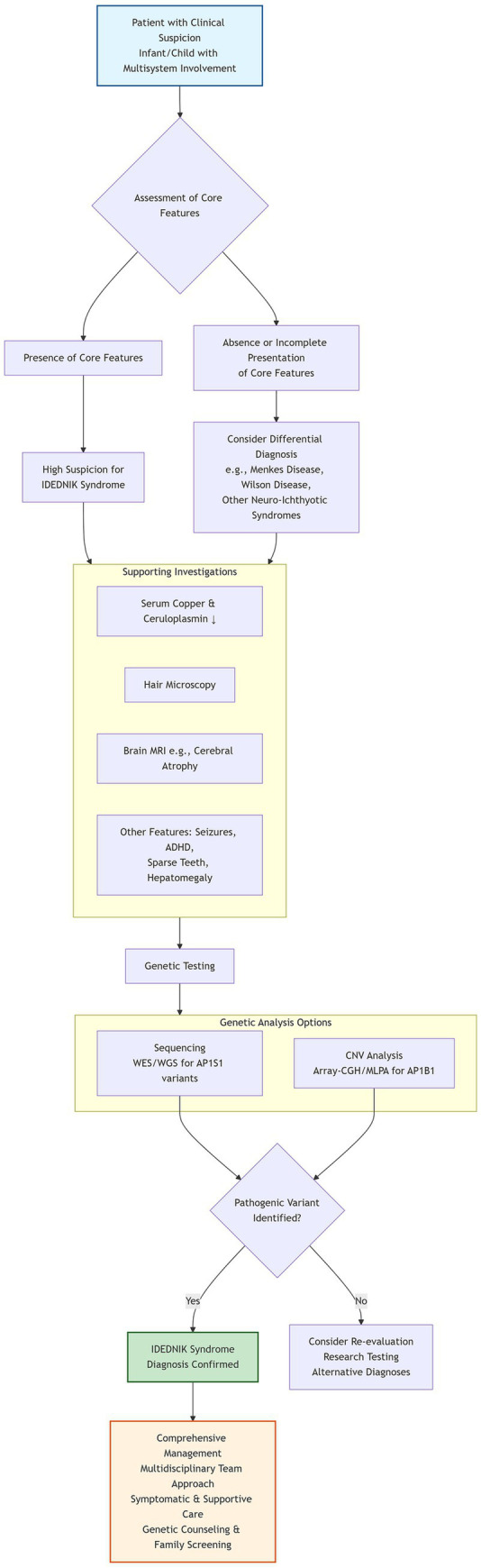
Diagnostic flowchart for IDEDNIK syndrome. This algorithm outlines the diagnostic approach for patients with suspected IDEDNIK syndrome. Core features refer to the characteristic manifestations represented by the IDEDNIK acronym: Intellectual Disability/Global Developmental Delay, Enteropathy (typically intractable diarrhea), Deafness (sensorineural), Peripheral Neuropathy, Ichthyosis, and Keratoderma. Other supporting features include additional manifestations such as seizures, attention deficit hyperactivity disorder (ADHD), sparse teeth, hepatomegaly, cerebral atrophy on MRI, and characteristic biochemical abnormalities (e.g., decreased serum copper and ceruloplasmin). Genetic testing should be pursued based on clinical suspicion and may include sequencing (whole-exome or whole-genome sequencing) to identify variants in *AP1S1* and/or copy number variant (CNV) analysis (array-CGH or MLPA) to detect exonic deletions/duplications in *AP1B1*. A definitive genetic diagnosis confirms IDEDNIK syndrome and enables initiation of multidisciplinary management. WES, whole-exome sequencing; WGS, whole-genome sequencing; Array-CGH, array comparative genomic hybridization; MLPA, multiplex ligation-dependent probe amplification.

The differential diagnosis is broad due to the multi-system nature of IDEDNIK syndrome. Key distinctions from other disorders (25–27) are summarized in Table 1. Additionally, we have developed a diagnostic flowchart, as detailed in Figure 1.

**Table 1:** Differential diagnosis of IDEDNIK syndrome among copper metabolism and neuro-ichthyotic disorders.

Disorder (Gene)	Key overlapping features with IDEDNIK	Distinguishing clinical/biochemical features
Menkes disease (*ATP7A*)	Intellectual disability, hypotonia, seizures, kinky/abnormal hair, low serum Cu/Cp	Pili torti on hair microscopy, connective tissue abnormalities, more severe neurodegeneration, typically earlier fatality.
Wilson’s disease (*ATP7B*)	Liver disease, neurological symptoms, Kayser-Fleischer rings (can be absent in young), occasionally low Cp.	Onset in childhood/adulthood, high urinary copper, effective with copper chelation therapy.
Huppke-Brendel syndrome (*SLC33A1*)	Congenital cataracts, hearing loss, hypotonia, low serum Cu/Cp.	Congenital cataracts, lack of prominent ichthyosis/enteropathy.
Aceruloplasminemia (*CP*)	Low ceruloplasmin, neurological symptoms.	Diabetes mellitus, retinal degeneration, high serum iron and ferritin.
CEDNIK syndrome (*SNAP29*)	Intellectual disability, ichthyosis, cerebral dysgenesis.	Palmoplantar keratoderma, microcephaly, agenesis of corpus callosum, no copper metabolism defects.
ARC syndrome (*VPS33B*/*VIPAR*)	Arthrogryposis, renal dysfunction, cholestasis, ichthyosis.	Arthrogryposis, renal tubular acidosis, platelet abnormalities, no primary copper metabolism defect.

Other conditions to consider include mitochondrial neurogastrointestinal encephalomyopathy (28), Zellweger syndrome (29), and other neuroichthyoses like Sjögren-Larsson syndrome or trichothiodystrophy (25).

## 5 Treatment, management, and future directions

At present, there is no disease-specific therapy for IDEDNIK syndrome. Management is primarily symptomatic and supportive, requiring a multidisciplinary approach to address multi-organ involvement and prevent complications.

### 5.1 Symptomatic and supportive management

#### 5.1.1 Zinc therapy

Zinc acetate has been used to reduce hepatic copper overload, with reported improvement in copper-related clinical features and biochemical abnormalities (12). It acts by inducing intestinal metallothionein, which sequesters copper and prevents its absorption. In regions where zinc acetate is unavailable, other zinc preparations (e.g., zinc gluconate) have been used anecdotally, with some reports of symptomatic improvement in diarrhea and pulmonary status, though biochemical correction may be incomplete (6).

#### 5.1.2 Skin care

Management includes low-dose oral retinoids and the use of various emollients with moisturizers and appropriate keratolytics (e.g., salicylic acid, urea, lactic acid) (30). Short-term use of topical corticosteroids or pimecrolimus ointment is recommended (31). Some patients experienced improvement in skin lesions following treatment with a compound cream containing triamcinolone acetonide, salicylic acid, and tar (32).

#### 5.1.3 Nutritional and gastrointestinal support

Dietary modifications are advised. Parenteral nutritional support or gastrostomy tube placement may be necessary for severe failure to thrive. Management of intractable diarrhea is challenging and may require fluid and electrolyte replacement.

#### 5.1.4 Neuromuscular and developmental management

Early intervention with physical, occupational, and speech therapy is essential. Seizures require appropriate antiepileptic drugs (AEDs) under neurological supervision; levetiracetam and sodium valproate have been used with some success (6). ADHD and other behavioral issues should be managed with standard therapies.

#### 5.1.5 Hearing and sensory rehabilitation

Hearing aids or cochlear implants may be considered for sensorineural hearing loss. Regular vision assessments are recommended.

#### 5.1.6 Monitoring and follow-up

Regular multidisciplinary assessments should include:

- Growth and nutrition: Anthropometric measurements, nutritional status evaluation.
- Dermatologic status: Skin lesion monitoring.
- Neurological function: Motor skills, tone, reflexes, seizure control.
- Sensory function: Hearing and vision assessments.
- Laboratory markers: Liver function tests, complete blood count, serum copper and ceruloplasmin. A summary of treatment approaches is detailed in Table 2.

**Table 2:** Summary of multidisciplinary management for IDEDNIK syndrome.

System/Aspect	Recommended management strategies
Copper metabolism	Oral zinc acetate (12) [or alternative zinc preparations (6)] to reduce copper absorption and hepatic load.
Dermatologic (Skin)	Emollients, keratolytics (e.g., salicylic acid, urea), low-dose oral retinoids (30); short-term topical corticosteroids or pimecrolimus (31); compound creams (e.g., with triamcinolone acetonide, salicylic acid, and tar) (32).
Gastrointestinal & nutritional	Dietary modifications; parenteral nutrition or gastrostomy tube for severe failure to thrive (6, 12); fluid/electrolyte replacement for diarrhea (11, 13).
Neurological & developmental	Antiepileptic drugs (e.g., levetiracetam, valproate) for seizures (6, 15); physical, occupational, and speech therapy; management of ADHD/behavioral issues (6).
Sensory (Hearing)	Hearing aids or cochlear implants for sensorineural hearing loss.
Monitoring & follow-up	Regular assessment of growth, nutritional status, liver function, serum copper/ceruloplasmin, and neurological/sensory function (12, 13).

### 5.2 Genetic counseling and testing

Relatives should undergo early genetic counseling to:

- Clarify autosomal recessive inheritance patterns.
- Assess recurrence risk for carrier parents.
- Discuss prenatal diagnostic options (e.g., chorionic villus sampling or amniocentesis for *AP1S1* and *AP1B1* testing, including CNV analysis).
- Consider preimplantation genetic diagnosis.

### 5.3 Emerging and future therapies

- Gene therapy: some rare genetic diseases have entered clinical trials for gene replacement or correction (33). While still in early stages for IDEDNIK syndrome, future application is plausible.
- Organoid technology: advances in organoid models raise the possibility of repairing or replacing affected organ tissues in the future, potentially improving survival and quality of life (6).

## 6 Prognosis

The prognosis of IDEDNIK syndrome remains poor. Deaths predominantly occur during early childhood, with contributing causes including severe diarrhea, feeding difficulties, and recurrent infections (6, 12, 31). Fewer than 50 cases have been reported worldwide, and long-term outcome data for survivors into later childhood or adulthood are extremely limited (12, 31). The phenotypic heterogeneity suggests that some genotypes may be associated with a less severe course (31), but more data is needed.

## 7 Summary and future directions

IDEDNIK syndrome is an extremely rare neurocutaneous genetic disorder resulting from pathogenic variants in *AP1S1* or *AP1B1*, which encode subunits of the AP-1 complex. Although the precise pathogenic mechanisms remain incompletely understood, current evidence implicates impaired intracellular protein trafficking and disrupted copper homeostasis.

Critical knowledge gaps and future research priorities include:

1. Elucidating pathogenesis: further studies are needed to fully unravel the trafficking defects beyond copper transporters and their link to the diverse clinical features.
2. Genotype–phenotype correlations: more reported cases with detailed genetic and clinical data are required to establish whether specific mutations in *AP1S1* or *AP1B1* correlate with disease severity or specific symptom clusters.
3. Natural history studies: prospective, international registries are essential to define the long-term clinical course, survival, and spectrum of disability in affected individuals.
4. Therapeutic development: research should focus on validating the efficacy of zinc therapy and exploring novel approaches, such as gene therapy or targeted molecular interventions, to correct the underlying trafficking defect.
5. Diagnostic refinement: increasing awareness of the full clinical spectrum, including newer features like sparse teeth (6), and the importance of comprehensive genetic testing (including CNV analysis for *AP1B1*) will improve diagnostic yield and ensure timely, accurate diagnosis for patients.
